# Long-Term Tracking of the Central Corneal Endothelial Mosaic

**DOI:** 10.1371/journal.pone.0088603

**Published:** 2014-03-13

**Authors:** Laura Gasser, Moritz Daniel, Thomas Reinhard, Daniel Böhringer

**Affiliations:** Eye Center, University Hospital Freiburg, Freiburg, Germany; University of Missouri-Columbia, United States of America

## Abstract

**Purpose:**

When comparing follow-up endothelial cell (EC) density measurements it is only possible to demonstrate cell loss in large cohorts or in pronounced cases due to the standard deviation of measurements. However, especially in clinical studies or refractive surgery patients, EC stability is an important factor. Thus we developed a computer program to achieve cell-by-cell alignment of conventional specular non-contact EC photographs. This is a pilot study to evaluate whether this new diagnostic technique is applicable in postoperative patients.

**Methods:**

Digitized endothelial cell photos of 30 eyes following implantation of a phakic posterior chamber intraocular lens for correction of high myopia were analyzed. All EC centroids were dotted on each image, and early and late follow-up pictures were automatically aligned on the basis of these centroids. The tracking results were confirmed via alternating image presentation of the corresponding image areas, and were reviewed for loss of individual cells. In addition, conventional EC density measurements were performed.

**Results:**

Mean time interval between first and second postoperative EC image was 1.4 years (range 3 months – 2.2 years), with early images taken at a median of 5.5 months after IOL implantation (lower/upper quartile 1.2–12.3 months). Extrapolated central EC density was 2812±500/mm^2^ at the first time point, and 2797±524/mm^2^ at the second time point. In 26 out of 30 image pairs, the EC mosaic was successfully retraced; cell loss in this area was excluded via photo flickering. Only in 4 image pairs, the EC mosaic could not be matched.

**Conclusions:**

We demonstrate that the corneal EC mosaic of clinical routine non-contact microscope pictures can be superimposed and compared on single cell level over time with our new computer based program. This new method is valuable to judge on EC stability even in small cohorts since it does not require mean values and standard deviations.

## Introduction

The endothelial cell (EC) layer of the cornea is crucial for its clarity - loss of ECs can lead to bullous keratopathy and the need for keratoplasty in the long run. Not only corneal diseases but also previous surgery can lead to irreversible EC loss and endothelial decompensation [1]. Thus it is not only necessary for clinicians to detect EC loss but also for clinical studies to reliably rule out chronic EC loss as a side effect of treatments. Especially for young refractive surgery patients who undergo phakic lens implantation, stability of EC density following lens implantation is an important safety factor.

When determining EC loss it is common practice to calculate the EC density in the central cornea at different time points. However, these density measurements vary [2], and thus large study groups would be necessary for statistical reasons in order to detect slight chronic EC loss. Nethertheless, slight loss can accumulate to a substantial EC loss over time and result in bullous keratopathy. Any method to diagnose EC stability in individual eyes would thus be of great benefit in the context of clinical trials on intraocular devices.

To overcome the problem that follow-up measurements show slightly different areas of the central EC mosaic and thus present slightly different cell counts, we wondered whether it would be possible to find the corresponding EC areas during follow-up measurements in the same eye, and then to concentrate on cell changes in these identical areas. The feasibility of this method to achieve cell-by-cell alignment of conventional specular non-contact EC photographs has beed described previously: We observed that the EC mosaic can be reliably retrieved in repeated EC photographs from the same day [3]. However, it is unclear whether this is also possible after longer follow-up intervals. Furthermore, previous intraocular surgery might impede EC tracking. Therefore, we herein investigate whether tracking the CE mosaic is possible after implantation of a phakic posterior chamber intraocular lens (pIOL) for correction of myopia.

## Methods

All eyes with digitized endothelial cell photos (n = 30) of a consecutive series of Epi.Lens-implantations for correction of high myopia (n = 40) were analyzed. Ten EC photographs from the Epi.Lens trial (The Epi.Lens clinical study was approved by the ethics committee of the University Freiburg, and participants provided written informed consent to participate in this study) had not been digitized directly and were not included in this study. All EC photographs had been acquired with the Robo Noncon SP 9000 microscope (Konan Medical, Japan). We manually selected early and late postoperative EC photos with good image quality and homogenous illumination for each eye. On each image, centroids of all ECs (i.e. the geometric centres of the two-dimensional EC hexagon) were marked automatically by the software, and only corrected manually as necessary. The software then used these centroid dot patterns to find matching image areas as described previously [3], slightly shifting or rotating the images as needed. Correct alignment of the two images was confirmed by two independent investigators via alternating image presentation (“photo flickering”) of the aligned pictures. During this “photo flickering”, the centroids were suppressed as not to distract the observers from the endothelial cell pattern. The aligned EC image pairs were compared and analyzed for loss of individual cells.

In addition, the EC density was conventionally assessed on each photo (“center to center method” [2]). We opposed diagnosis of stability by means of our new tracking method to conventional EC density measurements.

All analyses were performed with the R system [4].

## Results

The mean time interval between first and second postoperative image pair was 1.4 years (minimum 3 months, maximum 2.2 years). Our early images had been taken at a median of 5.5 months after pIOL implantation (lower/upper quartile 1.2–12.3 months).

In the conventional EC density measurements, all cells visible on the images were dotted (average of 190 ±32 marked cells) resulting in an extrapolated central EC density of 2812/mm^2^ (SD ±500) at the first time point, and 2797/mm^2^ (SD ±524) at the second time point. The difference in EC density between the first and the second follow-up date averaged −16/mm^2^ (minimum −450/mm^2^, maximum +407/mm^2^, SD ±200).

**Figure 1:**
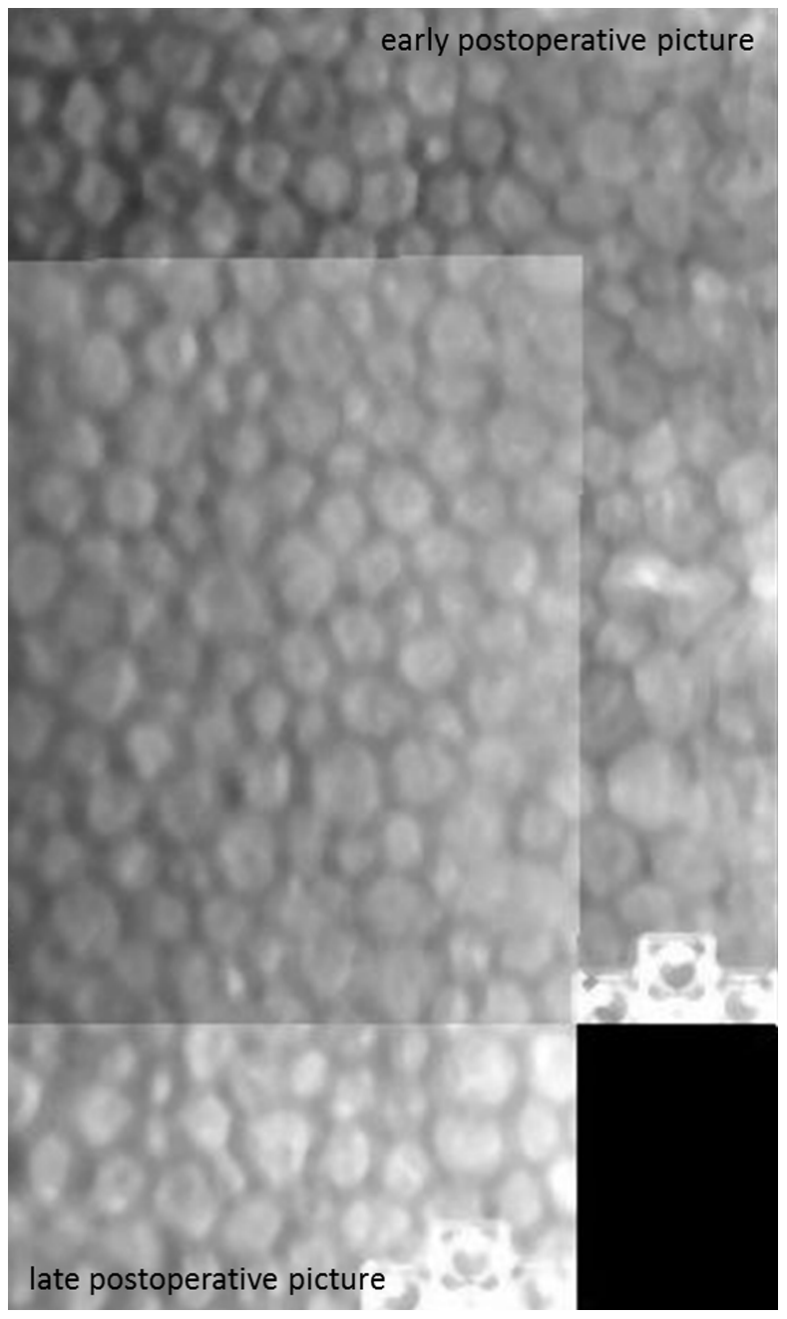
Matched and superimposed pair of early and late postoperative follow-up endothelial cell photographs.

**Figure 2:**
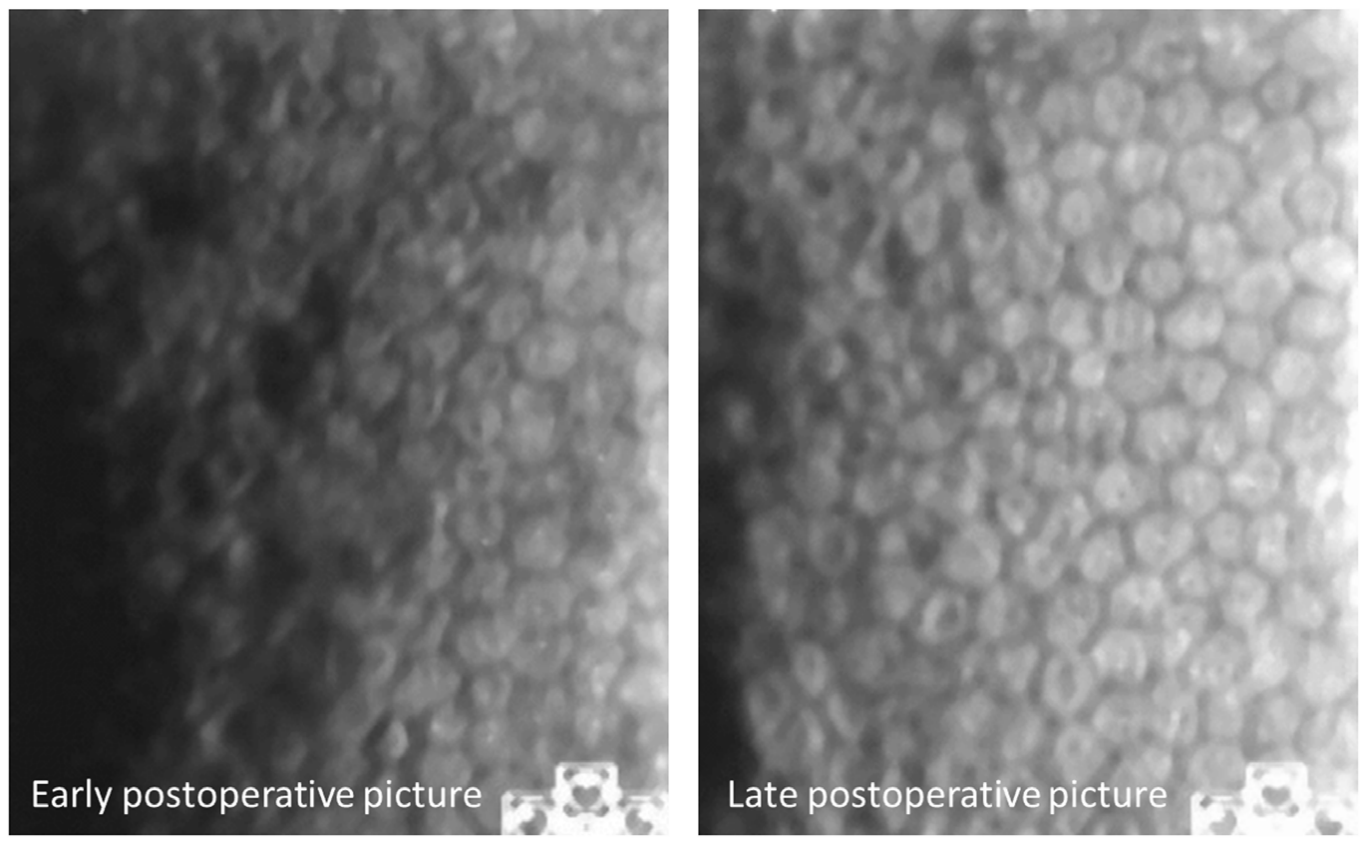
No successful match, probably due to poor illumination of early follow-up picture.

We managed to recover the EC mosaic in 26 of the 30 image pairs (87%). This means that individual ECs can robustly be retrieved after a mean follow-up of 1.4 years, even after intraocular surgery. Out of all cells on the photographs, no single cell loss was observed in 25 of 26 matched pairs (see Fig. 1). In one eye, however, focal cell loss was presumed because adjacent cells had slightly shifted. Out of the four pairs without a match, one image pair had a rather low image quality (only 91 cells marked compared to an average of 190 cells in high quality pictures of similar cell density) (see Fig. 2). The three others had 158–181 cells dotted. Cell density changes in these four pairs were −136/mm^2^, −111/mm^2^, +126/mm^2^ and −30/mm^2^, respectively.

## Discussion

Here, we report for the first time that the corneal EC mosaic can be traced between repeated measurements with non-contact microscopes used in clinical practice even several years after intraocular surgery. This can be used in clinical studies e.g. for evaluating EC changes following refractive surgery: We retrospectively evaluated postoperative EC changes by cell-by-cell alignment using routine EC photographs and compared the results with conventional EC density counts in a group of highly myopic patients who received a phakic, sulcus fixated intraocular lens. This is the first time that EC stability following surgery can be directly demonstrated for the central cornea in a small cohort – in contrast, with routine EC density measurements it is only possible to prove either a pronounced cell loss in a small cohort or smaller changes in ECs in large cohorts due to measurement deviations and statistical reasons.

Generally, our new tool is intended to verify cell stability and is thus useful for cohorts where no gross EC changes are anticipated. Contrary, the method is not designed for cohorts with known pronounced cell loss, since with a strongly altered EC mosaic no matches will be retrieved.

Our patient cohort comprised of the first patients who ever received a new type of phakic, sulcus fixated posterior chamber lens (Epi.Lens) [5] for correction of high myopia. Particularly in these otherwise healthy, young, refractive surgery patients, EC stability following lens implantation is important for long-term safety. Especially anterior chamber lenses have been associated with EC loss [6], in some cases leading to the complication of bullous keratopathy and the need for keratoplasty [7]. Phakic posterior chamber lenses on the other hand are rather associated with cataract formation as main complication, and endothelial changes are thought to be of a lesser extent following surgery [8]. However, small but nonetheless important changes in the EC density will not reach statistically significant levels in small cohorts of pilot studies due to the inaccuracy of mean values and standard deviations. Thus, small but important EC loss can easily be missed even in clinical studies looking at safety of intraocular devices, surgical techniques or potentially toxic substances.

Thus, we took a new approach by trying to confirm cell stability rather than looking at loss of cell density. The basic idea of this new technique is not to compare cell counts – but to compare images of the exact same area of cells and present them alternatingly (photo flickering), so that changes in the cell mosaic become apparent to the investigator. We have shown before that the principle of aligning repeated endothelial cell measurements on the same day is feasible [3]: Digitalized routine endothelial cell measurement pictures are automatically matched by our computer program (including possible rotation, sliding or scaling of the image), and the matched result is presented as a flicker-image pair for the examiner to easily compare the pictures and confirm endothelial cell stability.

We managed to retrace the endothelial cell picture in a high percentage of cases, and in all but one of these cases, all individual ECs remained intact. Thus, EC stability for this part of the central region was verified.

However, if no cell-by-cell alignment is achieved, several reasons are possible: 1. Endothelial cell loss (we believe that changes of the EC density outside the measured area might indirectly also alter the cell mosaic of the measured area by cell sliding to cover defect areas – this might lead to an (intended) failure to align the follow-up EC images, even when the cell loss might not be in the small area of the picture; 2. No overlap of the area of repeated EC measurements by malfixation, and 3. Poor picture quality.

**Figure 3:**
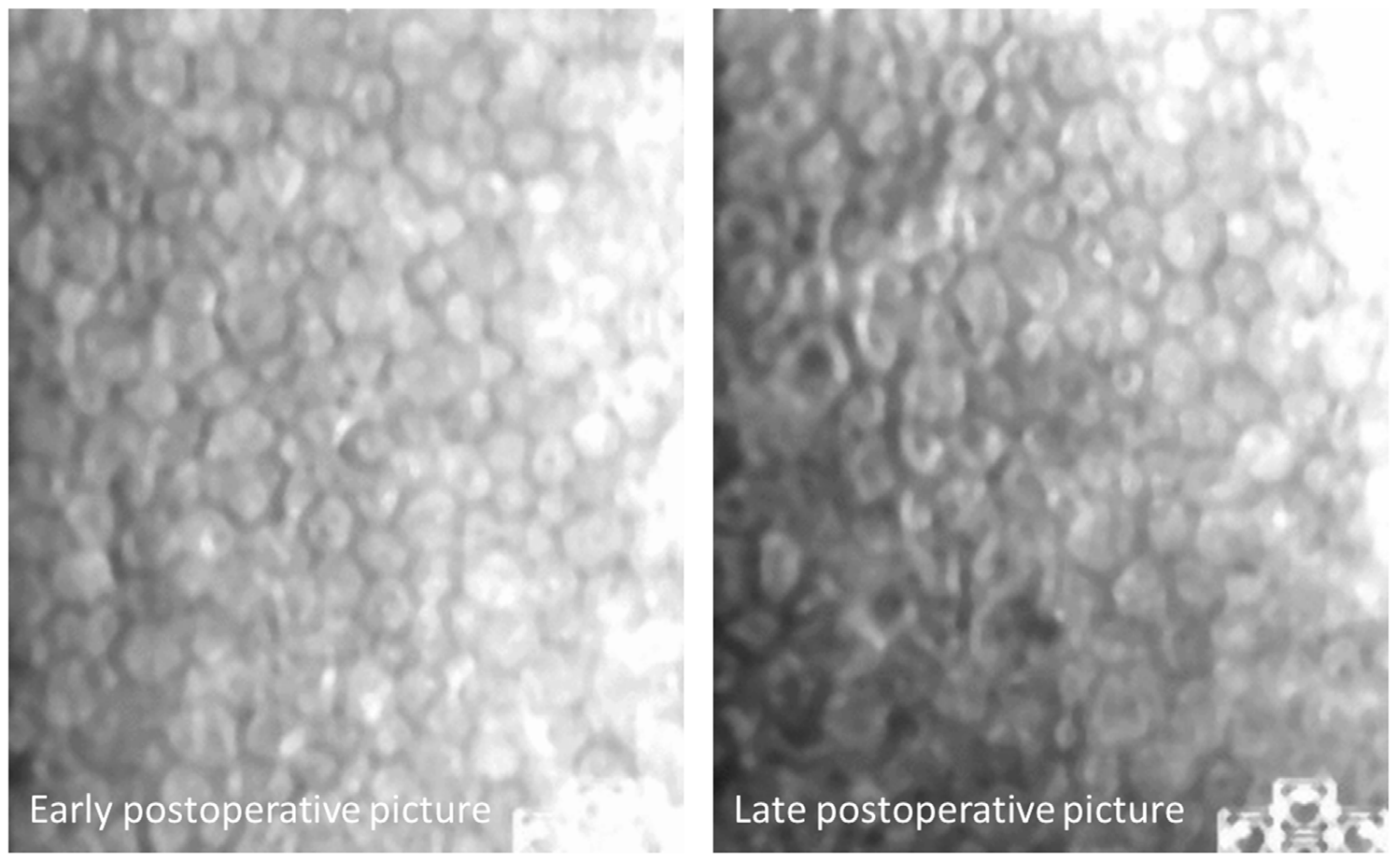
No successful match despite good image quality: No sufficient overlap (malfixation?) or cell loss? Comparison with conventional cell density measurement shows 2616 cells/mm^2^ vs. 2477 cells/mm^2^ in the later picture – taken together, cell loss cannot be excluded in this case.

In our study, we failed to align the pictures of 4 cases: In case 1, low picture quality (see fig. 2) might be the reason. In the other three eyes, no cell-by-cell alignment was achieved despite good picture quality (see fig. 3): In such a case we suggest to correlate two factors: misalignment and comparison of the endothelial cell density in these pictures. In these three cases, cell count changes ranged between −136/mm^2^ and +126/mm^2^. In case of pronounced loss of cell density, real cell loss is likely, but cannot be excluded in any of these three cases (see fig. 2).

There are limitations to this study: This is a pilot study designed to show feasibility of cell-by-cell alignment over time following implantation of an intraocular device. To judge on the safety of our implanted lens type, further analysis and longer follow-up will be necessary. In addition, preoperative pictures were not included, thus possible cell loss due to the intraocular device was tested, but not the surgical trauma during implantation.

As the case for all routine EC measurements, only a small fraction of the EC layer is analyzed. However, we suggest that our method might in fact be able to also detect cell changes adjacent to the picture by cell sliding to cover defects, thus “enlarging” the area taken into account.

To further optimize the rate of successful picture alignments despite imprecise fixation, it might be possible to digitally stitch together three overlapping repeated measurements for each time point to increase the total area of analysis.

Taken together, we believe to provide a useful tool adding to the information of EC changes in clinical studies. Routine EC measurements can be taken and analyzed by our computer program, for the first time directly confirming endothelial cell stability over time, even in small cohorts, rather than just statistically excluding cell loss in large cohorts.
